# Superspreading and heterogeneity in transmission of SARS, MERS, and COVID-19: A systematic review

**DOI:** 10.1016/j.csbj.2021.08.045

**Published:** 2021-09-01

**Authors:** Jingxuan Wang, Xiao Chen, Zihao Guo, Shi Zhao, Ziyue Huang, Zian Zhuang, Eliza Lai-yi Wong, Benny Chung-Ying Zee, Marc Ka Chun Chong, Maggie Haitian Wang, Eng Kiong Yeoh

**Affiliations:** a JC School of Public Health and Primary Care, The Chinese University of Hong Kong, Hong Kong Special Administrative Region, China; b School of Public Health, Zhejiang University, Hangzhou, China; c Shenzhen Research Institute, The Chinese University of Hong Kong, Shenzhen, China; d Mianyang Maternal and Child Health Care Hospital, Mianyang, China; e Department of Biostatistics, University of California Los Angeles Fielding School of Public Health, Los Angeles, CA, USA; f CUHK Institute of Health Equity, The Chinese University of Hong Kong, Hong Kong Special Administrative Region, China; g Centre for Health Systems and Policy Research, The Chinese University of Hong Kong, Hong Kong Special Administrative Region, China

**Keywords:** COVID-19, SARS, MERS, Superspreading, Transmission heterogeneity

## Abstract

**Background:**

Severe acute respiratory syndrome (SARS), Middle East respiratory syndrome (MERS), and coronavirus disease 2019 (COVID-19) have caused substantial public health burdens and global health threats. Understanding the superspreading potentials of these viruses are important for characterizing transmission patterns and informing strategic decision-making in disease control. This systematic review aimed to summarize the existing evidence on superspreading features and to compare the heterogeneity in transmission within and among various *betacoronavirus* epidemics of SARS, MERS and COVID-19.

**Methods:**

PubMed, MEDLINE, and Embase databases were extensively searched for original studies on the transmission heterogeneity of SARS, MERS, and COVID-19 published in English between January 1, 2003, and February 10, 2021. After screening the articles, we extracted data pertaining to the estimated dispersion parameter (*k*) which has been a commonly-used measurement for superspreading potential.

**Findings:**

We included a total of 60 estimates of transmission heterogeneity from 26 studies on outbreaks in 22 regions. The majority (90%) of the *k* estimates were small, with values less than 1, indicating an over-dispersed transmission pattern. The point estimates of *k* for SARS and MERS ranged from 0.12 to 0.20 and from 0.06 to 2.94, respectively. Among 45 estimates of individual-level transmission heterogeneity for COVID-19 from 17 articles, 91% were derived from Asian regions. The point estimates of *k* for COVID-19 ranged between 0.1 and 5.0.

**Conclusions:**

We detected a substantial over-dispersed transmission pattern in all three coronaviruses, while the *k* estimates varied by differences in study design and public health capacity. Our findings suggested that even with a reduced *R* value, the epidemic still has a high resurgence potential due to transmission heterogeneity.

## 1 Introduction

In the past two decades, coronavirus diseases have caused substantial public health burdens and global health threats. Between 2002 and 2004, over 8,000 cases and approximately 750 deaths caused by severe acute respiratory syndrome (SARS)-coronavirus (SARS-CoV) infection were observed [1]. Around 2013, Middle East respiratory syndrome (MERS)-coronavirus (MERS-CoV) emerged in the Middle East, causing 2,500 laboratory-confirmed cases and over 880 associated deaths by 2019 [2]. The ongoing coronavirus disease 2019 (COVID-19) pandemic, caused by SARS-CoV 2 (SARS-CoV-2), has resulted in more than 210 million cases and over 4.3 million deaths worldwide, as of August 10, 2021 [3].

Among the many important epidemiological parameters describing the transmission process of an infectious disease, the reproduction number (*R*), which is defined as the average number of secondary cases generated by a typical infectious individual [4], represents the transmission potential of an infectious pathogen at a population scale. However, *R* fails to reflect the heterogeneity in transmissibility among individuals, which is widely observed in coronaviruses [5]. Outbreaks involving an unusually large number of secondary cases are often seeded by only one or a few index cases, as observed in the COVID-19 [6], [7], [8], SARS [9], and MERS [10] epidemics. These phenomena are known as superspreading events (SSEs), and the initial source of infection is regarded as a super-spreader [11].

Theoretical models are important tools for characterizing and quantifying the heterogeneity in transmission, but have been implemented differently across studies. In some studies, standard compartment models (e.g., the classic susceptible-infectious-recovered model) were combined with superspreading compartments to characterize the transmission heterogeneity of SARS [12] and MERS outbreaks [13]. In contrast, some studies [14], [15], [16], [17] fitted the observed epidemiological data by a negative binomial (NB) distribution to quantify the superspreading potential. This approach was initially proposed by Lloyd-Smith *et al.* [18], in which the heterogeneity of infectiousness among individuals was quantified by estimating the dispersion parameter (*k*). Furthermore, *k* can also be estimated through phylogenetic analysis [19] or modeled as a latent variable in standard compartment models [20].

In general, a small *k* value indicates higher heterogeneity in transmission. If *k* is less than 1, the NB distribution has an exponential tail [21], which indicates that the transmission pattern is substantially over-dispersed. Different from the typical phenomenon, an over-dispersed transmission manifest the concept that a small proportion of people generate a large proportion of transmission. This phenomenon has also been described as the ‘20/80′ rule, which stipulates that 20% of the most infectious cases are responsible for 80% of the transmission (or secondary cases) [22]. Theoretically, when *k* is small and *R* is sufficiently large, the value of *k* approximates the proportion of the most infectious cases that generate 80% of the total transmissions [23]. Understanding transmission heterogeneity could provide key indications for public health strategies in disease control. When an epidemic exhibits a heterogeneous transmission pattern, that is, when *k* is small, even if the local situation is considered to be under control, with an *R* value of approximately 1, the epidemic still has a high resurgence potential [24].

Early studies evaluated the superspreading potential and transmission heterogeneity of coronaviruses during different periods and under different intervention measures [17], [25], [26]. However, a comprehensive summary of the superspreading potential of the three coronavirus diseases is lacking, and few studies have compared their transmission dispersiveness. Therefore, the aim of this study was to compare the superspreading potential of these diseases by systematic reviewing existing estimates of their transmission heterogeneity, thereby providing key intelligence for better intervention and infectious disease control.

## 2 Methods

This systematic review was conducted in strict accordance with the Cochrane collaboration guidelines and the Preferred Reporting Items for Systematic reviews and Meta-Analyses’ (PRISMA) guideline [27]. MEDLINE, Embase, and PubMed databases were searched for literature published between January 1, 2003, and February 10, 2021. The details of the search strategies and outcomes are presented in Supplementary Table S1. We supplemented these searches by consulting with content experts (SZ and MKCC) and by scanning the bibliographies of the identified articles. All articles were imported into EndNote (version X8, Thomson Reuters, Carlsbad, CA, USA), and duplicate studies were removed before further analysis.

### 2.1 Article selection criteria

Two reviewers (JW and ZH) independently identified eligible studies. Consensus was reached by referring to a third reviewer (XC) when opinions differed. All articles were screened by title and abstract, followed by the full text, to determine if the following pre-determined criteria were met: (**I1**) the study characterized the transmission heterogeneity of SARS, MERS, or COVID-19 in the human population; (**I2**) the article comprised peer-reviewed original research; and (**I3**) the values of the dispersion parameter (*k*) or the ‘20/80′ rule were explicitly and exactly reported. Studies were excluded if they had the following features: (**E1**) focused only on wild zoonotic transmission cycles; (**E2**) included patients without virological evidence of SARS-CoV, MERS-CoV, or SARS-CoV-2 infection; (**E3**) presented insufficient data or information to quantify the transmission heterogeneity; and (**E4**) was not published in English.

### 2.2 Data extraction

A standard data extraction form was used to extract information from the selected studies by three independent reviewers (JW, XC, and ZH). The following information was collected: **(INF1)** basic information of the study (i.e., the name of the first author, year of publication, investigation period, geographical region); **(INF2)** study population and settings (i.e., confirmed cases, cases in nosocomial setting [hospital clusters], younger vs. elderly cases, symptomatic vs. asymptomatic cases); **(INF3)** type of disease (i.e., SARS, MERS, COVID-19); **(INF4)** type of dataset used to generate the estimation (i.e., transmission pairs, cluster size, epidemic size, genome sequences); and **(INF5)** measurements of transmissibility and heterogeneity in transmission, (i.e., dispersion parameter [*k*], ‘20/80′ rule, reproduction number [*R*]).

### 2.3 Quality assessment

Two reviewers (XC and ZH) independently evaluated the quality of each included study using the Appraisal Tool for Cross-Sectional Studies (AXIS) scale [28]. There are 20 ‘Yes/No’ questions in the AXIS scale. Seven questions measure the quality of reporting and study design, and six questions measure the possible introduction of biases. A higher AXIS score indicates better study quality. A score of 16 points is considered as the cut-off to distinguish between high- and low-quality studies [29].

### 2.4 Calculating k in studies reporting the ‘20/80′ rule

We consistently used *k* to represent the superspreading potential. If an article did not explicitly report *k*, but reported *R* and the transmission distribution profiles in the form of the ‘20/80′ rule, we generated an estimation of *k* by using the framework proposed by Endo *et al.* [14], which has also been adopted in other studies [15]. For given values of *R* and the ‘20/80′ rule, the overdispersion parameter *k* is given by

$$1-P=\overset{X}{\underset{0}{\int }}NB\left(x;k,\;\frac{k}{R+k}\right)dx,$$

where *X* satisfies

$$1-Q=\;\frac{1}{R}\overset{X}{\underset{0}{\int }}x\;NB\left(x;k,\;\frac{k}{R+k}\right)dx.$$

Here, *P* is the expected proportion of the most infectious individuals responsible for *Q* of all transmissions. $NB\left(∙\right)$ represents the NB distribution for secondary cases, with mean *R* and dispersion parameter *k.* For studies reporting both the ‘20/80′ rule and *R*, the confidence intervals of *k* were constructed by adapting the posterior estimation of these parameter values to the above equations. For studies reporting the ‘20/80′ rule without *R*, a range of *k* was obtained by assuming an *R* ranging from 0.5 to 4.

## 3 Results

**Fig. 1:**
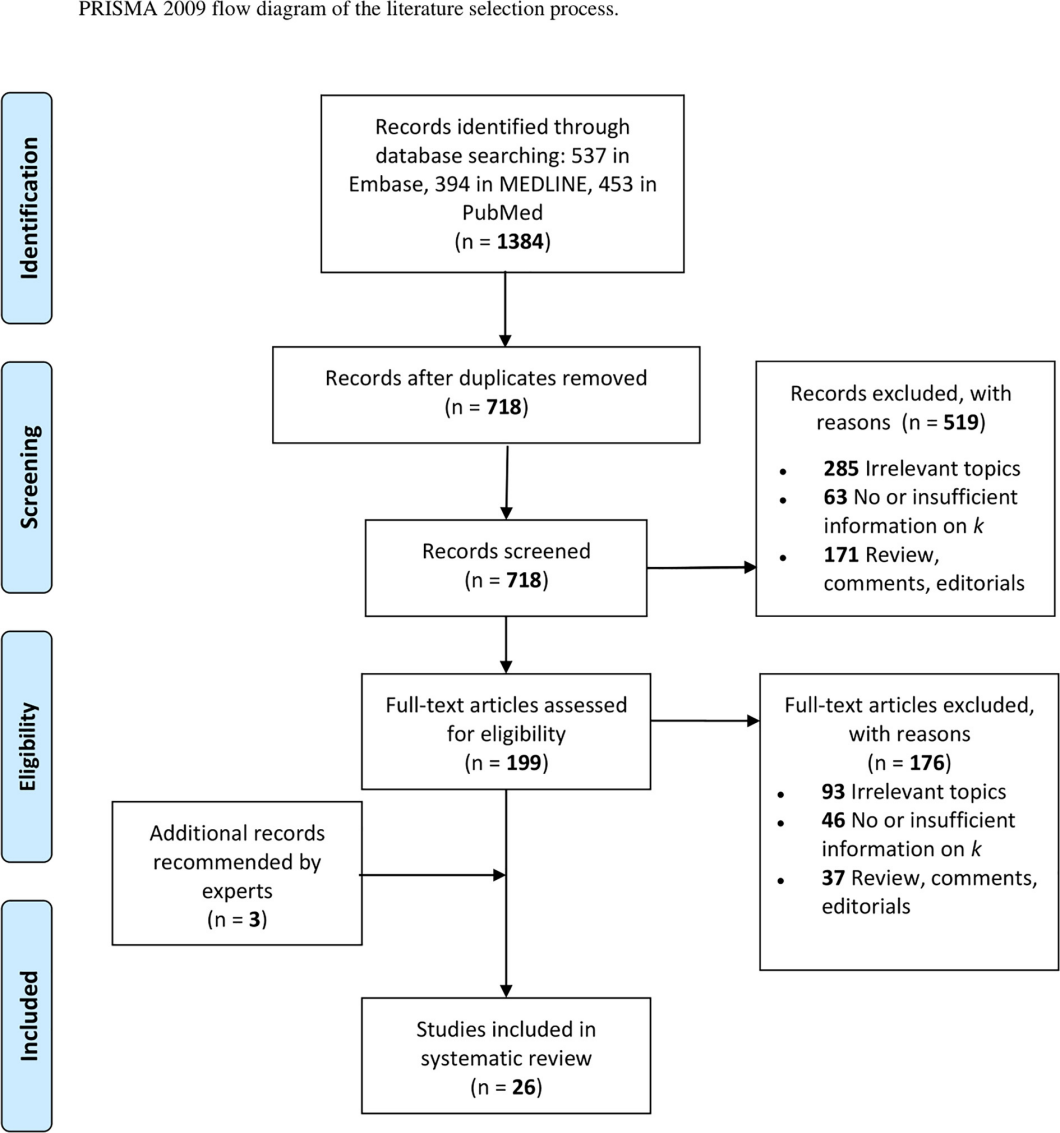
PRISMA 2009 flow diagram of the literature selection process.

In the literature searches of the Embase, MEDLINE, and PubMed databases, 1,384 records were identified (Fig. 1). There remained 718 articles after the removal of duplicates. After the initial screening, the full text of 199 articles was reviewed, resulting in the selection of 23 articles. Three additional articles were recommended by experts. Thus, 26 articles were finally selected. From these, 60 estimates of heterogeneity in transmission were included for further analysis. The AXIS quality scores of the included articles ranged from 14 to 19 points. Twenty-five articles (96.2%) met the criteria for a high-quality study. The detailed scores for each item on the AXIS are shown in Supplementary Table S2.

### 3.1 Characteristics of the included studies

The characteristics of the selected articles are listed in Supplementary Table S3. Among the 60 estimates of transmission heterogeneity, four (6.7%) from two studies estimated the dispersion parameter (*k*) of SARS [16], [18], 11 (18.3%) from eight articles estimated the *k* for MERS [16], [17], [30], [31], [32], [33], [34], [35], and 45 (75.0%) from 17 articles estimated the *k* for COVID-19 [14], [15], [19], [36], [37], [38], [39], [40], [41], [42], [43], [44], [45], [46], [47], [48], [49]. Forty estimates (66.7%) were based on transmission pair data (i.e., number of offspring cases generated by each index case), and four estimates were calculated using epidemic/cluster size data. The most common modelling method used to estimate *k* was a NB distribution, followed by phylogenetic analyses using genome sequence data.

### 3.2 Measurements of transmissibility and transmission heterogeneity

The dispersion parameter *k*, ‘20/80′ rule, and reproduction number were reviewed. Only five estimations (8.3%) [15], [17], [18], [38] reported all three measurements simultaneously. Twenty-five estimations (41.7%) reported only *k* without the other two measures [33], [41], [43], [46], [49], [50]. We conducted a secondary analysis to generate an estimated *k* value for seven articles that reported the ‘20/80′ rule [31], [32], [34], [35], [40], [43], [44] instead of a *k* value.

Overall, 54 of 60 estimates (90%) reported small values for *k*, with a scale of less than 1.

### 3.3 Estimated dispersion for SARS and MERS

**Fig. 2:**
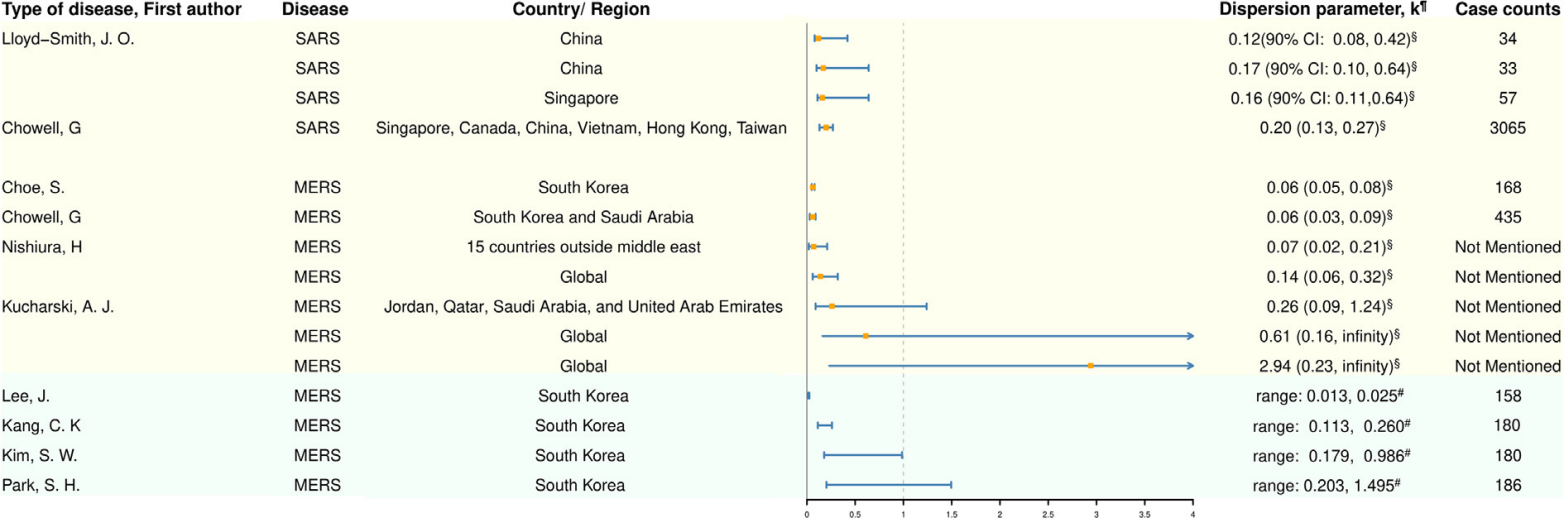
Estimated dispersion parameter (*k*) for SARS and MERS. **^¶^** Point estimates and 95% confidence intervals were reported, if not specified. ^§^Studies explicitly reported the *k* estimate. ^#^ Studies reported the ‘20/80′ rule, but not reproduction number or *k*. Only a range of *k* values were generated by using the method proposed in Endo *et al*[14], assuming a reproduction number ranges between 0.5 and 4.

The estimates of *k* for SARS and MERS are shown in Fig. 2. All estimates of *k* for SARS, and 90.9% (10/11) of those for MERS, were smaller than 1.

The *k* estimates for SARS ranged between 0.12 (90% confidence interval [CI]: 0.08, 0.42) in Beijing [18] to 0.20 (95% CI: 0.13, 0.27) in a global study [16], which assumed reproduction numbers of 1.88 [18] and 0.95 (95% CI: 0.67, 1.23) [28], respectively. Both estimates were derived from hospital clusters. Additionally, a study in Singapore reported an estimated *k* value of 0.16 (90% CI: 0.11, 0.64) based on confirmed cases regardless of transmission patterns [18].

For MERS, the point estimates of *k* ranged between 0.06 (95% CI: 0.03, 0.09) and 2.94 (95% CI: 0.23, infinity). Only one study [33] (9.1%) reported an estimated *k* larger than 1; this study used a likelihood-based inference applied to a global MERS dataset. For four studies [31], [32], [34], [35] that reported the ‘20/80′ rule, the converted *k* ranged between 0.013 and 1.495, and only one of these [35] had a lower bound of less than 1 (Fig. 2).

### 3.4 Estimated dispersion for COVID-19

The 45 estimates of *k* for COVID-19 had a wide range, from 0.009 (95% CI: 0.007, 0.348) in the United States (US) to 4.998 (95% CI: 0.003, infinity) in Israel. Over 91% of the *k* estimates (41/45) were derived from Asia, of which 23 estimates from seven studies were conducted in China [15], [19], [37], [42], [46], [47], [50]. The US was the only country studied outside of Asia [43].

**Fig. 3:**
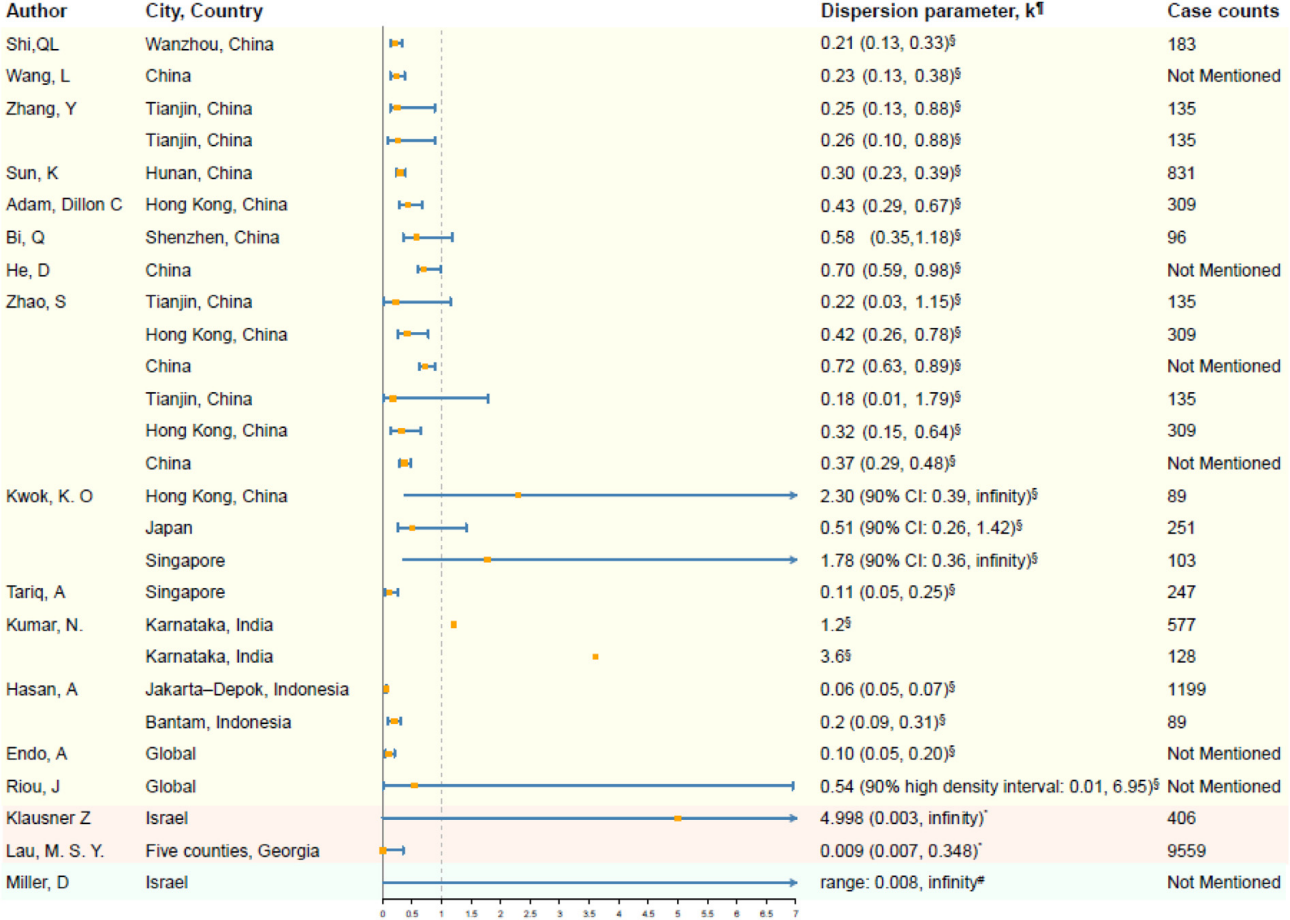
Estimated dispersion parameter (*k*) for COVID-19. ^¶^ Point estimates and 95% confidence intervals were reported (except for Endo *et al*, which is credible interval), if not specified. ^§^ Studies explicitly reported the *k* estimate. ^†^ Studies used non-truncated framework. ^‡^ Studies used truncated framework. * Studies reported the ‘20/80′ rule and reproduction number, but not *k*. We estimated *k* values and corresponding confidence intervals by using the method proposed in Endo *et al*[14]. ^#^ Studies reported the ‘20/80′ rule, but not *R* or *k*. Only a range of *k* values were generated by using the method proposed in Endo *et al*[14], assuming a reproduction number ranges between 0.5 and 4.

In total, 40% of the *k* estimates (18/45) were derived from subsets of data that focused on specific age ranges, transmission patterns, and generations (see Supplementary Table S3 for details). Additionally, all of these subgroup estimates were derived from studies conducted in China and the US. To avoid duplications and overweighting of these two countries, only the 27 estimates using data from the entire study population are shown in Fig. 3; among test, 22 estimates (81.5%) were smaller than 1. Regarding the five estimates larger than 1, two were from India [41], one was from Israel [40], and the other two, which used the number of offspring by each index case, were from Hong Kong [42] and Singapore [42].

## 4 Discussion

Understanding the potential for SSEs and transmission heterogeneity is helpful in determining the risks of over-dispersion in an epidemic and in informing effective infectious disease control measures. To our knowledge, this is the first systematic review to provide a comprehensive overview of the dispersion parameter, *k*, for SARS, MERS, and COVID-19. We included 60 estimates from 26 studies conducted in 22 countries/regions. Nearly half of the estimates pertained to transmission heterogeneity for COVID-19 in Asia. Most of the selected studies estimated the *k* value as lower than 1, suggesting a substantial over-dispersed transmission pattern in all three coronaviruses. Compared with that for SARS, wider ranges of estimated *k* values were observed for COVID-19 and MERS.

All studies from Indonesia, Japan, and the US reported a high potential for SSEs for COVID-19, while a low potential for SSEs for COVID-19 was reported in some studies from India, Hong Kong, and Israel. This may be partly explained by the combined effect of several extrinsic and intrinsic factors, such as the local public health capacity, study period, data type, and data quantity [13], [26]. For instance, the extremely large *k* for COVID-19 reported by a study conducted in Israel [40] may be attributable to a series of border control strategies and public health measures implemented even before confirmation of the first local case in Israel. These measures successfully prevented substantial transmission from imported cases, which were considered as the major initiator of SSEs in many other countries [15], [41], [45].

We also noted that studies conducted in the same region, but over different time periods, showed diverse outcomes. For example, an early study in Hong Kong that included data up to early March 2020 reported a large *k* estimate (2.3) [42], while a later study that included data up to April 28, 2020 reported a *k* less than 1 [15]. Similar differences in *k* estimates were also found in MERS studies conducted at different time periods [33]. These examples demonstrate the effect of study period on the *k* estimate.

The type of data also influenced the estimate of transmission heterogeneity. The data type used for calculating *k* mainly fell into two categories: ‘cluster size data’ and ‘transmission pair data’. The former comprises only information on the total cluster size, whereas the latter contains primary-secondary case pairs constructed by contact tracing. In general, we found that studies using cluster size data [14], [45] tended to estimate a higher heterogeneity than those using transmission pair data [19], [36], [37], [41], [46], [49]. This tendency was also noted in [15], in which the authors separately estimated *k* using both cluster size and transmission pair data collected during the same period. Although [14] pointed out that SSEs are more likely to be missed by contact tracing and the use of transmission pair data would overestimate *k,* cluster size data collected during an outbreak may have a relatively smaller sample size; hence, the resulting confidence interval for *k* is often much wider [15].

Regarding data quantity, previous studies suggested that when sample sizes are small, estimating *k* using the maximum likelihood (ML) method tends to overestimate *k*, while the risk of underestimating *k* is minimal [51]. This upward bias appears because small samples are less likely to cover a SSE [33]. Thus, for research that applied the ML method to small datasets, the results should be interpreted with caution.

During the study period covered by this review, very few *k* estimates for COVID-19 were based on data collected outside of Asia. This may be partly explained by different contract-tracing measures used within and outside Asia. While ‘backward tracing’, which traces the source cases who infected the index cases, were adopted by many Asian countries during the early phase of the COVID-19 epidemic (e.g. Japan and Singapore) [52], [53], contact tracing measures in non-Asia regions often targeted ‘downstream’ individuals who may have been infected by index cases (i.e., ‘forward tracing’) [54]. This may imply a lack of contact tracing and data reporting on identified transmission chains in the majority of regions beyond Asia. A previous stochastic modeling study suggested that backward tracing is much more effective in finding cases, especially when an over-dispersed transmission pattern arises [55]. As backward contact tracing measures have been gradually adopted in non-Asian countries, more non-Asian studies have estimated *k* and reported on the heterogeneity in COVID-19 transmission in the past few months [56], [57].

The substantial transmission heterogeneity reported in most of the reviewed articles may be partly attributable to the shared characteristics of all three coronaviruses in terms of the host, pathogen, and environment [58]. Heterogeneity in transmission can be explained by contact patterns and social behaviors, where super-spreaders often have notably higher numbers of contacts than others [59], [60], [61]. For example, majority of SSEs during the SARS and MERS outbreaks were linked to healthcare facilities that the index cases frequently visited [10], [31], [59], while COVID-19 SSEs have mainly occurred in social settings (e.g., bars, parties, and religious sites) [15], [62]. A recent modeling study further discussed the potential relationship between superspreading and social heterogeneity [23]. Furthermore, the large proportion of pre-symptomatic or asymptomatic cases of SARS-CoV-2 infection may escape from case ascertainment and maintain an active social life, implying difficulty in identifying the source cases of clusters and curtailing COVID-19 SSEs [63]. Some real-world studies on COVID-19 clusters in Japan and India found that a notable proportion of clusters in the community were due to pre-symptomatic or asymptomatic transmission [41], [64].

Higher virus loads and shedding in severe cases also enable a wider spread of disease to the mass population [59], [65], [66]. Furthermore, heterogeneity in the virus shedding profile would significantly contribute to shaping individual infectiousness. Recent studies have suggested that while the majority of COVID-19 cases are moderately infectious, as they barely expel the virus, highly infectious individuals are estimated to expel tens to thousands of infectious virus particles per minute [67], [68].

Environmental conditions are also crucial for driving SSEs. In the MERS outbreak in 2015, the overcrowding of healthcare facilities contributed to the emergence of SSEs [17]. Additionally, Yu *et al.* [69] verified that the occurrence of a superspreading outbreak during SARS was largely due to the airborne spread of the virus within and between residential buildings caused by the defects in drainage systems and air movement.

This study has some limitations. First, as the superspreading potential is context-dependent in nature, *meta*-analysis of *k* was not performed to avoid misinterpretation. Previous studies also discussed the strong influence of differences in the study period, data type, and statistical methods [46], [49]. Second, although this review provided a comprehensive picture of the superspreading potential of the three coronaviruses, the quality of evidence varied across diseases. For instance, compared with that for COVID-19, fewer studies were published on SARS and MERS, and a large proportion of *k* estimates for MERS were calculated backwardly in current study by using the ‘20/80′ rule. Third, among the studies on COVID-19, most *k* estimates were based on data from the early stages of the pandemic. More evidence is needed, considering the long-term nature of the pandemic and genetic mutations in pathogens. Lastly, this review considered 1 as the reference point for the *k* estimate. A few published estimates of *k* also indicated the risk of over-dispersion by comparing *k* with 1 [18], [33], considering that ‘*k* equals to 1′ leads to a sub-exponential tail in mathematical models; however, its linkage to epidemic dynamics or epidemiological interpretation remain unclear.

In conclusion, this systematic review provided a comprehensive overview of the superspreading potential and transmission heterogeneity of SARS, MERS, and COVID-19. We found that while the *k* estimates varied across studies due to the differences that arose in public health capacity and aspects of study design, the small *k* values among majority of the studies demonstrated a substantial over-dispersed transmission pattern in all three coronaviruses. Our findings suggested that even with a reduced *R* value, the epidemic of these three coronavirus diseases still has a high resurgence potential due to transmission heterogeneity.

**Funding**

This work is partially supported by CUHK grant [PIEF/Ph2/COVID/06, 4054600], Health and Medical Research Fund, the Food and Health Bureau, The Government of the Hong Kong Special Administrative Region [COVID190103, INF-CUHK-1], China and partially supported by the National Natural Science Foundation of China (NSFC) [31871340, 71974165].

## Declaration of Competing Interest

The authors declare that they have no known competing financial interests or personal relationships that could have appeared to influence the work reported in this paper.
