# Supplementary material for: Superspreading and heterogeneity in transmission of SARS, MERS, and COVID-19: A systematic review
Source: Comput Struct Biotechnol J. 2021 Sep 1;19:5039–46. doi: 10.1016/j.csbj.2021.08.045 (PMC8409018; doi:10.1016/j.csbj.2021.08.045)
Supplement: Supplementary data 1 [file mmc1.docx]

**Supplementary Tables and Figure Legends**

**Supplementary Table S1** Searching strategy for systematic review

**Supplementary Table S2**  Quality assessment of selected articles

**Supplementary Table S3** Characteristics of the selected articles

Supplementary Table S1 Searching strategy for systematic review

**Embase (n = 537)**

|  | [# ▲](http://ovidsp.dc1.ovid.com/sp-4.04.0a/ovidweb.cgi?&S=PLEIFPKMCBACEIPCKPBKHHGJAFDMAA00&Sort+Sets=descending) | **Searches** | **Number of identified records** |
| --- | --- | --- | --- |
|  | 1 | Middle east respiratory syndrome coronavirus.ti,ab,kw. | 1792 |
|  | 2 | MERS-CoV.ti,ab,kw. | 2746 |
|  | 3 | MERS.ti,ab,kw. | 6481 |
|  | 4 | Middle east respiratory syndrome.ti,ab,kw. | 2965 |
|  | 5 | Novel coronavirus.ti,ab,kw. | 7113 |
|  | 6 | Novel coronavirus 2012.ti,ab,kw. | 1 |
|  | 7 | Coronavirus.ti,ab,kw. | 48914 |
|  | 8 | SARS.ti,ab,kw. | 41629 |
|  | 9 | SARS-CoV.ti,ab,kw. | 32555 |
|  | 10 | Severe acute respiratory syndrome.ti,ab,kw. | 16399 |
|  | 11 | Atypical pneumonia.ti,ab,kw. | 1507 |
|  | 12 | SARS-CoV-2.ti,ab,kw. | 29670 |
|  | 13 | COVID-19.ti,ab,kw. | 84058 |
|  | 14 | 2019-nCoV.ti,ab,kw. | 1473 |
|  | 15 | Coronavirus 2019.ti,ab,kw. | 1238 |
|  | 16 | 2019 coronavirus.ti,ab,kw. | 406 |
|  | 17 | Wuhan coronavirus.ti,ab,kw. | 31 |
|  | 18 | Wuhan pneumonia.ti,ab,kw. | 20 |
|  | 19 | 1 or 2 or 3 or 4 or 5 or 6 or 7 or 8 or 9 or 10 or 11 or 12 or 13 or 14 or 15 or 16 or 17 or 18 | 116655 |
|  | 20 | Superspreader.ti,ab,kw. | 58 |
|  | 21 | Spreader.ti,ab,kw. | 881 |
|  | 22 | Superspreader event.ti,ab,kw. | 2 |
|  | 23 | Clusters of infection.ti,ab,kw. | 103 |
|  | 24 | Super-spreader.ti,ab,kw. | 58 |
|  | 25 | Super-spreader hosts.ti,ab,kw. | 1 |
|  | 26 | Super-spreading.ti,ab,kw. | 82 |
|  | 27 | Superspreading.ti,ab,kw. | 125 |
|  | 28 | Super spreading events.ti,ab,kw. | 41 |
|  | 29 | Super-spreader population.ti,ab,kw. | 0 |
|  | 30 | Variation in transmission.ti,ab,kw. | 120 |
|  | 31 | Transmission variation.ti,ab,kw. | 23 |
|  | 32 | Heterogeneity in transmission.ti,ab,kw. | 64 |
|  | 33 | Transmission heterogeneity.ti,ab,kw. | 75 |
|  | 34 | Transmission heterogeneities.ti,ab,kw. | 9 |
|  | 35 | Transmission potential.ti,ab,kw. | 1103 |
|  | 36 | Overdispersion.ti,ab,kw. | 968 |
|  | 37 | Dispersion parameter.ti,ab,kw. | 179 |
|  | 38 | Transmission dynamic.ti,ab,kw. | 257 |
|  | 39 | Transmission dynamics.ti,ab,kw. | 4276 |
|  | 40 | 20 or 21 or 22 or 23 or 24 or 25 or 26 or 27 or 28 or 29 or 30 or 31 or 32 or 33 or 34 or 35 or 36 or 37 or 38 or 39 | 8025 |
|  | 41 | 19 and 40 | 537 |

**MEDLINE (n = 394)**

|  | [# ▲](http://ovidsp.dc1.ovid.com/sp-4.04.0a/ovidweb.cgi?&S=PLEIFPKMCBACEIPCKPBKHHGJAFDMAA00&Sort+Sets=descending) | **Searches** | **Number of identified records** |
| --- | --- | --- | --- |
|  | 1 | Middle east respiratory syndrome coronavirus.ti,ab,kw. | 1336 |
|  | 2 | MERS-CoV.ti,ab,kw. | 1928 |
|  | 3 | MERS.ti,ab,kw. | 4468 |
|  | 4 | Middle east respiratory syndrome.ti,ab,kw. | 2102 |
|  | 5 | Novel coronavirus.ti,ab,kw. | 4244 |
|  | 6 | Novel coronavirus 2012.ti,ab,kw. | 1 |
|  | 7 | Coronavirus.ti,ab,kw. | 31806 |
|  | 8 | SARS.ti,ab,kw. | 24134 |
|  | 9 | SARS-CoV.ti,ab,kw. | 17696 |
|  | 10 | Severe acute respiratory syndrome.ti,ab,kw. | 10975 |
|  | 11 | Atypical pneumonia.ti,ab,kw. | 1256 |
|  | 12 | SARS-CoV-2.ti,ab,kw. | 17766 |
|  | 13 | COVID-19.ti,ab,kw. | 50428 |
|  | 14 | 2019-nCoV.ti,ab,kw. | 919 |
|  | 15 | Coronavirus 2019.ti,ab,kw. | 758 |
|  | 16 | 2019 coronavirus.ti,ab,kw. | 301 |
|  | 17 | Wuhan coronavirus.ti,ab,kw. | 19 |
|  | 18 | Wuhan pneumonia.ti,ab,kw. | 16 |
|  | 19 | 1 or 2 or 3 or 4 or 5 or 6 or 7 or 8 or 9 or 10 or 11 or 12 or 13 or 14 or 15 or 16 or 17 or 18 | 74563 |
|  | 20 | Superspreader.ti,ab,kw. | 40 |
|  | 21 | Spreader.ti,ab,kw. | 645 |
|  | 22 | Superspreader event.ti,ab,kw. | 3 |
|  | 23 | Clusters of infection.ti,ab,kw. | 80 |
|  | 24 | Super-spreader.ti,ab,kw. | 49 |
|  | 25 | Super-spreader hosts.ti,ab,kw. | 1 |
|  | 26 | Super-spreading.ti,ab,kw. | 59 |
|  | 27 | Superspreading.ti,ab,kw. | 70 |
|  | 28 | Super spreading events.ti,ab,kw. | 34 |
|  | 29 | Super-spreader population.ti,ab,kw. | 0 |
|  | 30 | Variation in transmission.ti,ab,kw. | 81 |
|  | 31 | Transmission variation.ti,ab,kw. | 15 |
|  | 32 | Heterogeneity in transmission.ti,ab,kw. | 43 |
|  | 33 | Transmission heterogeneity.ti,ab,kw. | 49 |
|  | 34 | Transmission heterogeneities.ti,ab,kw. | 6 |
|  | 35 | Transmission potential.ti,ab,kw. | 831 |
|  | 36 | Overdispersion.ti,ab,kw. | 704 |
|  | 37 | Dispersion parameter.ti,ab,kw. | 109 |
|  | 38 | Transmission dynamic.ti,ab,kw. | 150 |
|  | 39 | Transmission dynamics.ti,ab,kw. | 3159 |
|  | 40 | 20 or 21 or 22 or 23 or 24 or 25 or 26 or 27 or 28 or 29 or 30 or 31 or 32 or 33 or 34 or 35 or 36 or 37 or 38 or 39 | 5853 |
|  | 41 | 19 and 40 | 394 |

**PubMed (n = 453)**

|  | [# ▲](http://ovidsp.dc1.ovid.com/sp-4.04.0a/ovidweb.cgi?&S=PLEIFPKMCBACEIPCKPBKHHGJAFDMAA00&Sort+Sets=descending) | **Searches** | **Number of identified records** |
| --- | --- | --- | --- |
|  | 1 | Middle east respiratory syndrome coronavirus [Title/Abstract] | 1747 |
|  | 2 | MERS-CoV [Title/Abstract] | 2706 |
|  | 3 | MERS [Title/Abstract] | 5560 |
|  | 4 | Middle east respiratory syndrome [Title/Abstract] | 2929 |
|  | 5 | Novel coronavirus [Title/Abstract] | 7996 |
|  | 6 | “Novel coronavirus 2012” [Title/Abstract] | 0 |
|  | 7 | Coronavirus [Title/Abstract] | 53522 |
|  | 8 | SARS [Title/Abstract] | 46035 |
|  | 9 | SARS-CoV [Title/Abstract] | 38262 |
|  | 10 | Severe acute respiratory syndrome [Title/Abstract] | 18486 |
|  | 11 | Atypical pneumonia [Title/Abstract] | 657 |
|  | 12 | SARS-CoV-2 [Title/Abstract] | 35601 |
|  | 13 | COVID-19 [Title/Abstract] | 10011 |
|  | 14 | 2019-nCoV [Title/Abstract] | 1534 |
|  | 15 | Coronavirus 2019 [Title/Abstract] | 1376 |
|  | 16 | 2019 coronavirus [Title/Abstract] | 468 |
|  | 17 | Wuhan coronavirus [Title/Abstract] | 32 |
|  | 18 | Wuhan pneumonia [Title/Abstract] | 17 |
|  | 19 | 1 or 2 or 3 or 4 or 5 or 6 or 7 or 8 or 9 or 10 or 11 or 12 or 13 or 14 or 15 or 16 or 17 or 18 | 126792 |
|  | 20 | Superspreader [Title/Abstract] | 99 |
|  | 21 | Spreader [Title/Abstract] | 673 |
|  | 22 | “Superspreader event” [Title/Abstract] | 2 |
|  | 23 | “Clusters of infection” [Title/Abstract] | 0 |
|  | 24 | Super-spreader [Title/Abstract] | 69 |
|  | 25 | “Super-spreader hosts” [Title/Abstract] | 0 |
|  | 26 | Super-spreading [Title/Abstract] | 81 |
|  | 27 | Superspreading [Title/Abstract] | 203 |
|  | 28 | “Super spreading events” [Title/Abstract] | 43 |
|  | 29 | “Super-spreader population” [Title/Abstract] | 0 |
|  | 30 | “Variation in transmission” [Title/Abstract] | 0 |
|  | 31 | Transmission variation [Title/Abstract] | 15 |
|  | 32 | “Heterogeneity in transmission” [Title/Abstract] | 50 |
|  | 33 | Transmission heterogeneity [Title/Abstract] | 52 |
|  | 34 | Transmission heterogeneities [Title/Abstract] | 7 |
|  | 35 | Transmission potential [Title/Abstract] | 814 |
|  | 36 | Overdispersion [Title/Abstract] | 860 |
|  | 37 | Dispersion parameter [Title/Abstract] | 138 |
|  | 38 | Transmission dynamic [Title/Abstract] | 197 |
|  | 39 | Transmission dynamics [Title/Abstract] | 3545 |
|  | 40 | 20 or 21 or 22 or 23 or 24 or 25 or 26 or 27 or 28 or 29 or 30 or 31 or 32 or 33 or 34 or 35 or 36 or 37 or 38 or 39 | 6392 |
|  | 41 | 19 and 40  Filter to humans | 453 |

Supplementary Table S2 Quality assessment of selected articles

| **Author** | **Introduction** | **Methods** | | | | | | | | | | **Results** | | | | | **Discussion** | | **Other** | |
| --- | --- | --- | --- | --- | --- | --- | --- | --- | --- | --- | --- | --- | --- | --- | --- | --- | --- | --- | --- | --- |
|  | 1 | 2 | 3 | 4 | 5 | 6 | 7 | 8 | 9 | 10 | 11 | 12 | 13 | 14 | 15 | 16 | 17 | 18 | 19 | 20 |
| Sun, K^1^ | Y | Y | N | Y | Y | Y | Y | Y | Y | Y | Y | Y | Y | Y | Y | Y | Y | Y | N | Y |
| Adam, Dillon C^2^ | Y | Y | N | Y | Y | Y | Y | Y | Y | Y | Y | Y | Y | Y | Y | Y | Y | Y | N | Y |
| Bi, Q^3^ | Y | Y | N | Y | Y | Y | Y | Y | Y | Y | Y | Y | Y | Y | Y | Y | Y | Y | N | Y |
| Endo, A^4^ | Y | Y | Y | Y | Y | Y | Y | Y | Y | Y | Y | Y | Y | Y | Y | Y | Y | Y | N | N |
| He, D^5^ | Y | Y | N | Y | Y | Y | Y | Y | Y | Y | Y | Y | Y | Y | N | Y | Y | N | N | Y |
| Hasan, A^6^ | Y | Y | N | Y | Y | Y | Y | Y | Y | N | Y | Y | Y | Y | N | Y | Y | N | N | N |
| Riou, J^7^ | Y | Y | Y | Y | Y | Y | Y | Y | Y | Y | Y | Y | Y | Y | N | Y | Y | Y | N | N |
| Klausner Z^8^ | Y | Y | Y | Y | Y | Y | Y | Y | Y | Y | Y | Y | Y | Y | N | Y | Y | N | N | Y |
| Kumar, N.^9^ | Y | Y | Y | Y | Y | Y | Y | Y | Y | Y | Y | Y | Y | Y | N | Y | Y | Y | N | N |
| Kwok, K. O^10^ | Y | Y | Y | Y | Y | Y | Y | Y | Y | Y | Y | Y | Y | Y | N | Y | Y | N | N | N |
| Lau, M. S. Y.^11^ | Y | Y | Y | Y | Y | Y | Y | Y | Y | Y | Y | Y | Y | Y | Y | Y | Y | Y | N | N |
| Miller, D^12^ | Y | Y | N | Y | Y | Y | Y | Y | Y | Y | Y | Y | Y | Y | Y | Y | Y | N | N | Y |
| Tariq, A^13^ | Y | Y | Y | Y | Y | Y | Y | Y | Y | Y | Y | Y | Y | Y | Y | Y | Y | Y | N | Y |
| Wang, L^14^ | Y | Y | N | Y | Y | Y | Y | Y | Y | Y | Y | Y | Y | Y | N | Y | Y | N | N | N |
| Zhao, S^15^ | Y | Y | N | Y | Y | Y | Y | Y | Y | Y | Y | Y | Y | Y | N | Y | Y | Y | N | Y |
| Zhang, Y^16^ | Y | Y | Y | Y | Y | Y | Y | Y | Y | Y | Y | Y | Y | Y | N | Y | Y | Y | N | N |
| Shi,QL^17^ | Y | Y | Y | Y | Y | Y | Y | Y | Y | Y | Y | Y | Y | Y | Y | Y | Y | Y | N | Y |
| Choe, S.^18^ | Y | Y | Y | Y | Y | Y | Y | Y | Y | Y | Y | Y | Y | Y | N | Y | Y | Y | N | N |
| Chowell, G^19^ | Y | Y | N | Y | Y | Y | Y | Y | Y | Y | Y | Y | Y | Y | N | Y | Y | Y | N | Y |
| Nishiura, H^20^ | Y | Y | Y | Y | Y | Y | Y | Y | Y | Y | Y | Y | Y | Y | N | Y | Y | Y | N | N |
| Kang, C. K^21^ | Y | Y | Y | Y | Y | Y | Y | Y | Y | Y | Y | Y | Y | Y | N | Y | Y | Y | N | Y |
| Kim, S. W.^22^ | Y | Y | Y | Y | Y | Y | Y | Y | Y | Y | Y | Y | Y | Y | N | Y | Y | Y | N | N |
| Kucharski, A. J.^23^ | Y | Y | Y | Y | Y | Y | Y | Y | Y | Y | Y | Y | Y | Y | N | Y | Y | Y | N | N |
| Lee, J.^24^ | Y | Y | Y | Y | Y | Y | Y | Y | Y | Y | Y | Y | Y | Y | N | Y | Y | N | N | N |
| Park, S. H.^25^ | Y | Y | Y | Y | Y | Y | Y | Y | Y | Y | Y | Y | Y | Y | Y | Y | Y | Y | N | N |
| Lloyd-Smith, J. O.^26^ | Y | Y | Y | Y | Y | Y | Y | Y | Y | Y | Y | Y | Y | Y | N | Y | Y | Y | N | Y |

Y, Yes; N, Not; NM, not mentioned.

According to quality of cross-sectional studies (AXIS) scale (Downes MJ, Brennan ML, Williams HC, Dean RS. Development of a critical appraisal tool to assess the quality of cross-sectional studies (AXIS). *BMJ open* 2016; **6**(12): e011458.):

*Introduction*

1. Were the aims/objectives of the study clear?

*Methods*

2. Was the study design appropriate for the stated aim(s)?

3. Was the sample size justified?

4. Was the target/reference population clearly defined? (Is it clear who the research was about?)

5. Was the sample frame taken from an appropriate population base so that it closely represented the target/reference population under investigation?

6. Was the selection process likely to select subjects/participants that were representative of the target/reference population under investigation?

7. Were measures undertaken to address and categorise non-responders?

8. Were the risk factor and outcome variables measured appropriate to the aims of the study?

9. Were the risk factor and outcome variables measured correctly using instruments/ measurements that had been trialled, piloted or published previously?

10. Is it clear what was used to determined statistical significance and/or precision estimates? (eg, p values, cis)

11. Were the methods (including statistical methods) sufficiently described to enable them to be repeated?

*Results*

12. Were the basic data adequately described?

13. Does the response rate raise concerns about non-response bias?

14. If appropriate, was information about non-responders described?

15. Were the results internally consistent?

16. Were the results for the analyses described in the methods, presented?

*Discussion*

17. Were the authors’ discussions and conclusions justified by the results?

18. Were the limitations of the study discussed?

*Other*

19. Were there any funding sources or conflicts of interest that may affect the authors’ interpretation of the results?

20. Was ethical approval or consent of participants attained?

References

1. Sun K, Wang W, Gao L, et al. Transmission heterogeneities, kinetics, and controllability of SARS-CoV-2. *Science (New York, NY).* 2020.

2. Adam DC, Wu P, Wong JY, et al. Clustering and superspreading potential of SARS-CoV-2 infections in Hong Kong. *Nature medicine.* 2020;26(11):1714-1719.

3. Bi Q, Wu Y, Mei S, et al. Epidemiology and transmission of COVID-19 in 391 cases and 1286 of their close contacts in Shenzhen, China: a retrospective cohort study. *The Lancet Infectious Diseases.* 2020;20(8):911-919.

4. Endo A. Estimating the overdispersion in COVID-19 transmission using outbreak sizes outside China. *Wellcome Open Research.* 2020;5.

5. He D, Zhao S, Xu X, et al. Low dispersion in the infectiousness of COVID-19 cases implies difficulty in control. *BMC public health.* 2020;20(1):1558.

6. Hasan A, Susanto H, Kasim MF, et al. Superspreading in early transmissions of COVID-19 in Indonesia. *Scientific reports.* 2020;10(1):22386.

7. Riou J, Althaus CL. Pattern of early human-to-human transmission of Wuhan 2019 novel coronavirus (2019-nCoV), December 2019 to January 2020. *Eurosurveillance.* 2020;25(4):2000058.

8. Klausner Z, Fattal E, Hirsch E, Shapira SC. A single holiday was the turning point of the COVID-19 policy of Israel. *International Journal of Infectious Diseases.* 2020;101:368-373.

9. Kumar N, Shahul Hameed SK, Babu GR, et al. Descriptive epidemiology of SARS-CoV-2 infection in Karnataka state, South India: Transmission dynamics of symptomatic vs. asymptomatic infections. *EClinicalMedicine.* 2021:100717.

10. Kwok KO, Chan HHH, Huang Y, et al. Inferring super-spreading from transmission clusters of COVID-19 in Hong Kong, Japan, and Singapore. *Journal of Hospital Infection.* 2020;105(4):682-685.

11. Lau MSY, Grenfell B, Thomas M, Bryan M, Nelson K, Lopman B. Characterizing superspreading events and age-specific infectiousness of SARS-CoV-2 transmission in Georgia, USA. *Proceedings of the National Academy of Sciences of the United States of America.* 2020;117(36):22430-22435.

12. Miller D, Martin MA, Harel N, et al. Full genome viral sequences inform patterns of SARS-CoV-2 spread into and within Israel. *Nature communications.* 2020;11(1):5518.

13. Tariq A, Lee Y, Roosa K, et al. Real-time monitoring the transmission potential of COVID-19 in Singapore, March 2020. *BMC Medicine.* 2020;18(1):166.

14. Wang L, Didelot X, Yang J, et al. Inference of person-to-person transmission of COVID-19 reveals hidden super-spreading events during the early outbreak phase. *Nature Communications.* 2020;11(1):5006.

15. Zhao S, Shen M, Musa SS, et al. Inferencing superspreading potential using zero-truncated negative binomial model: exemplification with COVID-19. *BMC Medical Research Methodology.* 2021;21(1):1-8.

16. Zhang Y, Li Y, Wang L, Li M, Zhou X. Evaluating transmission heterogeneity and super-spreading event of COVID-19 in a metropolis of China. *International Journal of Environmental Research and Public Health.* 2020;17(10):3705.

17. Shi Q, Hu Y, Peng B, et al. Effective control of SARS-CoV-2 transmission in Wanzhou, China. *Nature medicine.* 2021;27(1):86-93.

18. Choe S, Kim HS, Lee S. Exploration of superspreading events in 2015 mers-cov outbreak in korea by branching process models. *International Journal of Environmental Research and Public Health.* 2020;17(17):1-14.

19. Chowell G, Abdirizak F, Lee S, et al. Transmission characteristics of MERS and SARS in the healthcare setting: A comparative study. *BMC Medicine.* 2015;13(1):210.

20. Nishiura H, Endo A, Saitoh M, et al. Identifying determinants of heterogeneous transmission dynamics of the middle east respiratory syndrome (MERS) outbreak in the Republic of Korea, 2015: A Retrospective epidemiological analysis. *BMJ Open.* 2016;6(2):e009936.

21. Kang CK, Song KH, Choe PG, et al. Clinical and Epidemiologic Characteristics of Spreaders of Middle East Respiratory Syndrome Coronavirus during the 2015 Outbreak in Korea. *Journal of Korean medical science.* 2017;32(5):744-749.

22. Kim SW, Park JW, Jung HD, et al. Risk factors for transmission of Middle East respiratory syndrome coronavirus infection during the 2015 outbreak in South Korea. *Clinical Infectious Diseases.* 2017;64(5):551-557.

23. Kucharski AJ, Althaus CL. The role of superspreading in middle east respiratory syndrome coronavirus (MERS-CoV) transmission. *Eurosurveillance.* 2015;20(25):14-18.

24. Lee J, Chowell G, Jung E. A dynamic compartmental model for the Middle East respiratory syndrome outbreak in the Republic of Korea: A retrospective analysis on control interventions and superspreading events. *Journal of Theoretical Biology.* 2016;408:118-126.

25. Park SH, Kim Y, Jung Y, et al. Outbreaks of middle east respiratory syndrome in two hospitals initiated by a single patient in Daejeon, South Korea. *Infection and Chemotherapy.* 2016;48(2):99-107.

26. Lloyd-Smith JO, Schreiber SJ, Kopp PE, Getz WM. Superspreading and the effect of individual variation on disease emergence. *Nature.* 2005;438(7066):355-359.

Supplementary Table S3 Characteristics of the selected articles

Please see another supplement excel table
